# Molecular Epidemiology and Species Diversity of Tick-Borne Pathogens of Animals in Egypt: A Systematic Review and Meta-Analysis

**DOI:** 10.3390/pathogens11080912

**Published:** 2022-08-14

**Authors:** El-Sayed El-Alfy, Ibrahim Abbas, Hanadi B. Baghdadi, Shimaa Abd El-Salam El-Sayed, Shengwei Ji, Mohamed Abdo Rizk

**Affiliations:** 1 Parasitology Department, Faculty of Veterinary Medicine, Mansoura University, Mansoura 35516, Egypt; 2 Biology Department, College of Science, Imam Abdulrahman Bin Faisal University, Dammam 31113, Saudi Arabia; 3 Basic and Applied Scientific Research Center (BASRC), Imam Abdulrahman Bin Faisal University, Dammam 31113, Saudi Arabia; 4 National Research Center for Protozoan Diseases, Obihiro University of Agriculture and Veterinary Medicine, Inada-Cho, Obihiro 080-8555, Hokkaido, Japan; 5 Department of Biochemistry and Chemistry of Nutrition, Faculty of Veterinary Medicine, Mansoura University, Mansoura 35516, Egypt; 6 Department of Internal Medicine, Infectious and Fish Diseases, Faculty of Veterinary Medicine, Mansoura University, Mansoura 35516, Egypt

**Keywords:** tick-borne diseases, Egypt, molecular, *Anaplasma*, *Babesia*, *Theileria*, *Coxiella burnetii*, *Ehrlichia*, *Rickettsia*, *Borrelia*, meta-analysis

## Abstract

Ticks and tick-borne pathogens (TTBPs) are listed among the most serious concerns harming Egyptian livestock’s productivity. Several reports on tick-borne pathogens (TBPs) from various geographical regions in the country were published. However, data on the molecular characterization of TBPs are the most beneficial for understanding the epidemiology of this important group of pathogens. In this study, we present the first meta-analysis on the molecular epidemiology and species diversity of TBPs infecting animals in Egypt. All published studies on TBPs were systematically collected from various databases (PubMed, Scopus, ScienceDirect, the Egyptian Knowledge Bank, and Google Scholar). Data from eligible papers were extracted and subjected to various analyses. Seventy-eight studies were found to be eligible for inclusion. Furthermore, ticks infesting animals that were molecularly screened for their associated pathogens were also included in this study to display high species diversity and underline the high infection risk to animals. *Theileria annulata* was used as parasite model of TBPs to study the genetic diversity and transmission dynamics across different governorates of Egypt. This study extends cross-comparisons between all published molecular data on TBPs in Egypt and provides resources from Egyptian data in order to better understand parasite epidemiology, species diversity, and disease outcome as well as the development and implementation of prevention and control methods for public health, veterinary care practitioners, and animal owners all over the country.

## 1. Introduction

Tick-borne diseases (TBD) are important factors that constrain the development of livestock industries worldwide and can cause losses estimated to be billions of dollars for farmers annually [1,2]. Phenotypic traits are proven to have limited taxonomic significance in identification and delimitation of various species during microscopical examination [3,4]. The use of molecular diagnostic tools in studying tick-borne agents has increased in recent decades because of its high sensitivity and accuracy [5,6,7,8]. With the advancement of molecular biology, new species, strains, or genetic variants of microorganisms are being discovered in ticks all over the world, and the list of potential tick-borne infections is growing [9].

Egypt’s population is rapidly growing. The estimated population in 2020 was 102.3 million with an annual rate of population change of 2.03% (United Nations population estimates and projections; https://population.un.org (accessed on 1 June 2022). The local animal population exceeded 18 million, comprising 5.1 million cattle, 3.7 million water buffaloes, 5.4 million sheep, 4 million goats, 120,000 camels, and 85,000 horses [10,11]. Food security is one of the challenges facing the world due to the fast-rising human population, and the global prevalence of undernourished people increased drastically between 2019 and 2020, owing primarily to the COVID-19 pandemic [12]. Stakeholders were urged to adopt a One Health approach to designing and implementing livestock policies and investments, particularly in dealing with emerging and re-emerging animal diseases that, if left uncontrolled, could endanger the development trajectory of the entire livestock sector [10].

Ticks, which are vectors of more pathogens than any other group of invertebrates, have become a growing focus of attention among the different arthropods capable of transmitting pathogens that can cause serious diseases in animals and humans [13,14]. While the Middle East and North Africa (MENA) have suitable climates and favorable conditions for the propagation and spread of ticks, reports on TTBPs in this area are scarce [15]. Despite recent advances in the characterization and taxonomic justification of various tick-borne pathogens infecting animals in Egypt, there has never been a comprehensive analysis for the epidemiology of TTBPs. Our study is the first to conduct a systematic review and meta-analysis determining the prevalence, based on pooled estimates, and species diversity of various TBPs infecting animals in Egypt and to evaluate the associated risk factors, including the impact of geographic distribution as well as pathogens in infesting ticks.

## 2. Data Collection and Analysis

### 2.1. Searching Strategy

The databases PubMed, Scopus, and ScienceDirect were searched for studies in English published until May 2022 on TTBPs infecting animals in Egypt. The search was refined by the article type of research articles. Various keywords were used for the search, including ticks, tick-borne diseases, *Babesia,* babesiosis, *Theileria*, theileriosis, *Anaplasma*, anaplasmosis, *Coxiella burnetii*, Q fever, *Ehrlichia*, ehrlichiosis, *Rickettsia*, rickettsioses, *Borrelia*, borreliosis, CCHF, and Crimean–Congo haemorrhagic fever. The keywords were used in combinations with the animal species (cattle, buffalo, sheep, goats, camels, equines, horses, donkeys, and dogs) and detection method (molecular and PCR) as well as “Egypt” (Table 1). To combine the entry terms, the Boolean operators “OR” and “AND” were used. In addition, the Egyptian Knowledge Bank’s website (http://www.ekb.eg accessed on 23 May 2022) was searched to collect papers from Egypt published in local journals. To ensure the successful collection of data and the inclusion of the full data of relevant papers rather than abstracts, the Google Scholar search engine was employed. The same keywords were used in all databases.

### 2.2. Eligibility Criteria

**Figure 1:**
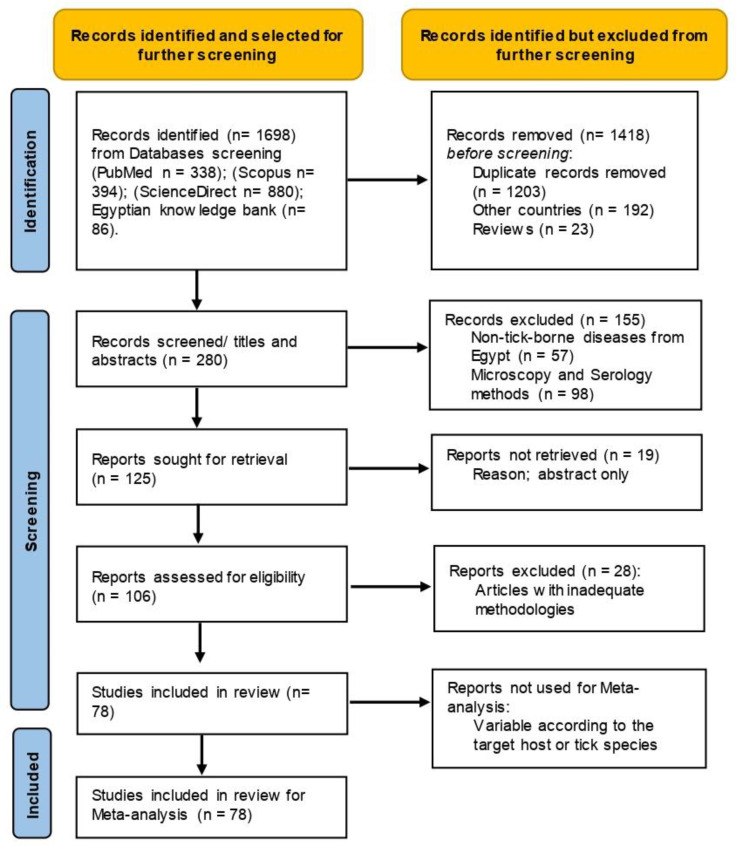
Flow diagram established according to PRISMA guidelines and displaying the search and selection methodology.

The collected publications were screened for inclusion independently, and studies with disagreements were discussed (Figure 1). Studies were considered eligible for inclusion in this review when (1) the study found PCR-positive samples for TBPs in cattle, buffaloes, sheep, goats, camels, horses, donkeys, and dogs from Egypt; (2) the study defined the number of examined animals and number of positives; (3) the study stated information on ticks collected from animals and described the tick pools at least to the genus level based on morphological or molecular characteristics and molecularly identified their harboured TBPs; and (4) molecular studies with sequenced isolates that were deposited on GenBank. Studies that did not meet these criteria were considered ineligible; for example, we did not include (1) studies on TBPs in countries other than Egypt; (2) studies on non-tick-borne pathogens from Egypt; (3) studies with non-original contributions, e.g., review, book chapters, and seminars; (4) studies with inadequate methodologies; and (5) studies using microscopy and serology for the detection of TBPs.

### 2.3. Data Extraction

Data from eligible studies on TBPs infections of animals were extracted and organized in a Microsoft Excel^®^ spreadsheet, and any disagreement was resolved by consensus. The following information was extracted whenever possible: study subregion, sample size, number of positives, type of PCR used, genetic markers, TBPs detected, and accession numbers of the sequenced isolates. The authors of the included studies were not contacted for further information. Data conversions were applied to determine the positives of certain genera (e.g., *Babesia*) by the subtraction of the mixed species’ positive samples.

### 2.4. Meta-Analysis

The tabulated data in the Excel spreadsheets were used for various meta-analyses conducted in our study using the software Open Meta [Analyst] [16]. All analyses were conducted based on a 95% confidence interval. The prevalence for various TBPs were estimated as “pooled estimates”, employing the random effects model based on the DerSimonian-Laird method. The heterogeneity among the included studies was calculated based on the *I^2^* statistic, and the heterogeneity values were considered high when *I^2^* exceeded 50%. Subgroup analyses were conducted to investigate the prevalence variation in relation to the species diversity. Publication bias was not assessed in our study because it was not considered relevant for prevalence studies [17].

### 2.5. Molecular Analysis

The included studies were screened to obtain the accession numbers of isolates that were sequenced based on various gene markers. The nucleotide sequences of the obtained accession numbers were collected from the website of the National Center for Biotechnology (http://www.ncbi.nlm.nih.gov accessed on 13 May 2022). Additional information on the locations of the isolates were also extracted. The sequences were aligned with ClustalW software and carefully checked to confirm that they were all in the reading frame. A few isolates of TBPs that have been identified based on various genetic markers were collected (Tables S1–S5). This was not the case for *Theileria annulata*; an adequate number of partial Tams-1 and 18SrRNA nucleotide sections were found suitable for establishing the phylogenetic analysis (Table 2). The collected nucleotide sections were aligned, trimmed from both ends, and stored in a FASTA format that was used to construct the phylogenetic trees using the software MEGA6. The nucleotide substitution models with the best fit to the data set (lowest BIC) were chosen. The evolutionary history was deduced using the maximum likelihood method, which was based on the Tamura 3-parameter method and was modelled using the gamma distribution for 18SrNA gene sequences. The Hasegawa–Kishino–Yano (HKY) model was used for Tams-1 gene sequences. The same software was then used to convert the Tams-1 sequences into the Nexus format [18], which was used for establishing the haplotype networks using the minimum spanning model of the software PopArt 1.7 (Population Analysis with Reticulate Trees). The networks were developed in relation to the isolates’ governorates of origin [19].

## 3. Eligible Studies on TBPs in Animals in Egypt

**Figure 2:**
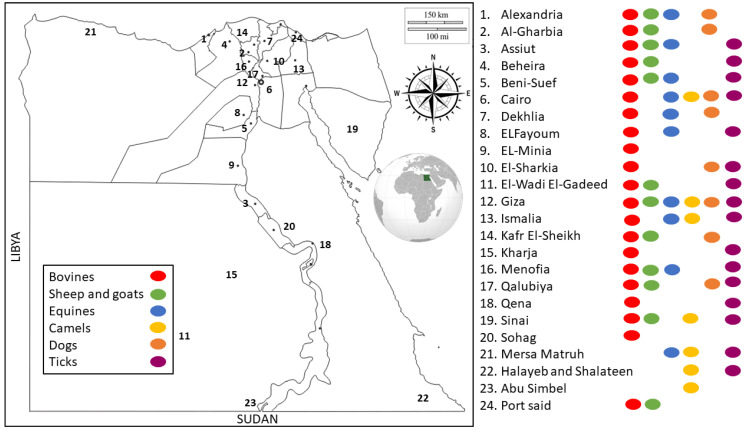
Distribution of molecular studies on TBPs of animals in different locations of Egypt. Key numbers on the map represent the distributions of molecular studies across different locations. Key dots represent the distributions of molecular studies according to different hosts combined with the distributions across different locations in Egypt.

Egypt has a unique location in the northeastern corner of Africa and southwestern area of Asia. Egypt is known for having a hot, dry climate throughout the country, and the summer temperatures are high, particularly in Upper Egypt, creating a suitable environment for various tick species [24,33]. A total of 78 studies from 24 locations all over Egypt (Figure 2) were reviewed, of which several detected TBPs infecting more than one host species. The included studies were categorized based on different hosts into 37 studies on bovines, 15 on sheep and goats, 9 on equines, 12 on dromedary camels, 11 on dogs, and 22 on ticks. In general, *Babesia*, *Theileria*, and *Anaplasma* were the most frequently tested TBPs.

## 4. TBPs in Cattle and Buffaloes in Egypt

Five of the thirty-seven molecular studies on bovines were not used for meta-analysis; the included data on those five were distinguished between cattle and buffaloes, or the number of positives was not clearly specified. Therefore, 32 studies were included, comprising 23 studies on cattle only, 1 study on buffaloes only, and 8 studies on both cattle and buffaloes. These studies molecularly tested 7213 cattle and 626 buffaloes for various TBPs (Table S1).

The data concerning the estimated pooled prevalence for various TBPs infecting cattle are summarized in Table 3. In total, 14 data sets describing *Babesia* infections in 3203 cattle were revealed during our database search, and 525 cases were found to be infected, resulting in a pooled prevalence of 16.0% (95% CI, 10.9–21.0%). Two *Babesia* spp. were frequently detected and displayed similar prevalences: *Babesia bigemina* (10.1%, CI, 6.3–13.8%) and *Babesia bovis* (9.5%, CI, 6.0–13.0%). A few datasets detected other species, e.g., *Babesia ovis* (7.3%) and *Babesia occultans* (0.3%). *Theileria* infections were the most frequently tested TBPs in cattle; 17 data sets tested 4620 cattle, and 1324 were found to be infected, giving rise to a pooled global prevalence of 36.0% (95% CI, 23.4–48.7%). Of the species detected, *T. annulata* was the predominant (30.8%), whereas a much lower prevalence was estimated for *Theileria orientalis* (3.0%). For *Anaplasma* infections, we collected 9 data sets that tested 1745 cattle, and 510 animals were found to be infected, resulting in the highest pooled prevalence (43.9%, CI, 4.8–83.1%) among TBPs infecting cattle. Likewise, *Anaplasma* displayed the greatest species diversity among cattle TBPs; several *Anaplasma* species were identified, including *Anaplasma marginale* (21.2%), *Anaplasma centrale* (1.4%), *Anaplasma platys*-like (8.3%), *Anaplasma platys* (8.4%), *Anaplasma phagocytophilum* (15.0%), and *Anaplasma ovis* (3.4%). It is noteworthy that in 2020 and 2021, *Anaplasma* infections outnumbered *Babesia* and *Theileria* infections in many cattle farms in Egypt (personal communication with various field veterinarians). However, the prevalence of the variations among the three common TBPs (*Babesia*, *Theileria*, and *Anaplasma*) infecting cattle were statistically insignificant (*p* value = 0.1960). Other miscellaneous TBPs that infect cattle were detected in lower prevalences, including *Bartonella* spp. (2.6%), *Borrelia* spp. (2.9%), *Coxiella burnetti* (7.2%), and *Rickettsia* sp. (1.1%).

Although the population of water buffaloes in Egypt is not much different than that of cattle, buffaloes have received little attention concerning TBPs. Similar to the TBPs in cattle, *Anaplasma* species were the most prevalent TBPs in buffaloes with a pooled prevalence of 26.9% (95% CI, 7.3–61.1%), and *A. marginale*, *A. platys*-like, and *A. platys* were the identified species (Table 4). The other TBPs detected in buffaloes displayed minor prevalences, e.g., *Babesia* species (*B. bigemina* and *B. bovis*) had a pooled prevalence of 3.6% (95% CI, 0.6–6.6%). Many field veterinarians in Egypt rely on combined conjunctivitis–lymphadenopathy as a specific symptom to diagnose chronic theileriosis in buffaloes. Based on personal communications, the disease is common in Egypt particularly during summer in 2020 or 2021. However, the estimated pooled prevalence for *Theileria* infections in buffaloes did not exceed 1.0%. A possible explanation for this very low prevalence in comparison to cattle (36.0%) is the limited number of tested buffaloes (247). It is noteworthy that many other pathogens can cause eye infections in buffaloes, particularly *Moraxella bovis*, which may lead to disease misdiagnosis. The low detection rate of piroplasms in water buffaloes may be attributed to their wallowing in muddy waters to maintain their body temperature, together with their thick hide, which contributes to lower tick attachment [34,35,36]. *Bartonella* species were also detected in buffaloes and expressed a higher prevalence (5.0%) than they did in cattle (2.6%).

Anaplasmosis (primarily caused by *A. marginale* and *A. centrale*), babesiosis (*B. bovis*, *B. bigemina*, and *Babesia divergens*), and theileriosis (*T. annulata*, *Theileria parva*, and *T. orientalis* complex) affect bovines worldwide, causing significant economic losses to the cattle industry, especially in the tropics and subtropics [37,38,39]. Thus, the frequent detection of these parasites from bovines in Egypt is alarming and requires the establishment of effective surveillance and control strategies. *Anaplasma marginale* is the most prevalent among TBPs in buffaloes (37.5%) and the second most prevalent in cattle (21.2%) in Egypt (after *T. annulata*). This parasite is also the most prevalent tick-borne pathogen globally in bovines, causing a mild to severe hemolytic disease with considerable economic loss [1,40].

## 5. TBPs in Sheep and Goats in Egypt

TBPs are not popular among small ruminant producers in Egypt, most likely due to the restricted resultant economic loss, in comparison with the various viral and bacterial diseases that are highly prevalent in sheep in Egypt. Fourteen studies were found that detailed the prevalence of TBPs in 1286 sheep and 263 goats (Table S2), and we included 6 data sets that described *Babesia* and *Theileria* infections in sheep with estimated pooled prevalences of 3.8% and 11.0%, respectively (Table 5). *Anaplasma* infections were also the most prevalent (16.1%, CI, 6.6–23.5%) in sheep and were investigated in four data sets, encompassing 599 animals. Other TBPs detected in sheep have displayed variable prevalences: *Bartonella* spp. (3.1%, CI, 3.3–9.6%), *Borrelia* spp. (3.4%, CI, 1.2–8.1%), and *Rickettsia* spp. (13.7%, CI, 12.1–39.6%). Notably, six data sets described *C. burnetti* infections in 309 sheep, and 94 animals were found positive, giving rise to a very high estimated pooled prevalence (45.3%, CI, 9.5–81.2%) (Table 5). Moreover, a diverse fauna of TBPs were identified in sheep, including various species of the genus *Babesia* (*B. bovis*, *B. bigemina*, and *B. ovis*), the genus *Theileria* (*T. annulata*, *Theileria ovis*, and *Theileria lestoquardi*), and the genus *Anaplasma* (*A. marginale*, *A. ovis*, *A. phagocytophilum*, *A. platys*, and *A. platys-like*). *Babesia ovis* and *T. lestoquardi* are the most pathogenic tick-borne haemoparasites in small ruminants worldwide [41].

Meanwhile, the data on TBPs in goats in Egypt are less informative since very few data sets (*n* = 4) were found. Four TBPs were investigated, including *Theileria* (50.0%), *C. burnetti* (29.4%), *Babesia* (16.7%), and *Bartonella* (2.0%) (Table 6). Q fever is a globally transmitted zoonotic infection caused by the intracellular Gram-negative bacterium *C. burnetii* [42]. Excretion of *C. burnetii* in tick faeces and saliva is widely reported, and the prevalence of *C. burnetii* in ticks from various bioclimatic zones and socioeconomic contexts suggests their potential role in the epidemiology of Q fever [43]. Although the molecular data indicated a high prevalence of Q fever in sheep and goats in Egypt, some of examined samples were seropositive and/or from aborted animals. While the high prevalence of *C. burnetti* is suggestive of the potential role of sheep and goats in the transmission of Q fever to people in Egypt, serosurveys from humans in Egypt are scarce [44,45,46]. Furthermore, molecular and serological data show that Q fever may play a role in sheep and goat abortions [45,47,48].

## 6. TBPs in Equines in Egypt

Nine studies that tested 855 horses and 546 donkeys were used in the meta-analyses conducted to estimate the pooled prevalence for various TBPs infecting equines in Egypt (Table S3). *Theileria* spp. were most prevalent in horses (34.1%, 95% CI, 12.9–55.3%) and donkeys (30.6%, 95% CI, 14.0–47.2%). *Theileria equi* and *Theileria haneyi* were identified in both horses and donkeys. Moreover, *Theileria* sp. Africa were detected in horses, whereas *T. ovis* were found in donkeys (Table 7 and Table 8). Two data sets described Babesiosis (*Babesia caballi*) in horses and donkeys, with pooled prevalences of 9.8% (CI,−7.8–27.5%) and 7.2% (CI,−7.2–21.5%), respectively. *Bartonella* spp. were also identified in horses (0.8%) and donkeys (5.1%); meanwhile, infection with *Anaplasma* spp. (*A. marginale* and *A. ovis*) was detected only in donkeys (26.7%). Equine piroplasmosis is an important tick-borne disease caused by the hemoprotozoan parasites *T. equi* and *B. caballi*, resulting in major economic losses to the equine industry [49,50,51].

## 7. TBPs in Dromedary Camels in Egypt

The dromedary (*Camelus dromedarius*), also referred to as the Arabian camel, dromedary camel, or one-humped camel, is a large even-toed ungulate that belongs to the family Camelus. In the Old World region, the domesticated dromedary is typically found in semi-arid to arid areas, primarily in Africa and the Arabian Peninsula, though there is also a sizable feral population in Australia [52,53].

Camels can host a wide range of very diverse TBPs. However, a few studies (*n* = 11) on dromedaries in Egypt that tested 1268 animals were found during the database search and determined to be suitable for the meta-analysis (Table S4). In general, high TBPs’ prevalence was detected, regardless of the limited number of datasets. Various species of *Babesia* (11.0%), *Theileria* (71.8%), and *Anaplasmsa* (40.5%) as well as C. *burnetti* (20.8%) and *Rickettsia* spp. (31.9%) were identified in the tested dromedaries in Egypt (Table 9). Of note, the TBPs detected in camels were more highly diverse than those of any other animal species (Table 9). The zoonotic species *Babesia microti* was interestingly identified in the blood of 17 out of 142 camels in one study. *Babesia microti* infects humans and is considered to be an important transfusion-transmitted infectious agent. Between 2010 and 2014, the parasite caused 4 out of 15 deaths associated with transfusion-transmitted infections in the United States [54]. The zoogeographical range of ticks and the diseases they transmit are limited by host movements and climatic variables [55,56]. In Egypt, significant numbers of animals are imported to compensate the gap in the livestock industry. All imported cattle are slaughtered in quarantine stations’ facilities. Camels are imported from various countries in East Africa and may be transferred to slaughterhouses or to various animal markets after being released from the quarantine. The Birqash market near Cairo is Africa’s biggest camel market. Between 2012 and 2015, a total of 762,291 camels were legally imported into Egypt from Sudan (79.4%) and Ethiopia (20.6%) [57]. Egypt obtains camels from Sudan, Somalia, Ethiopia, Eritrea, and Kenya by way of Ethiopia [58,59]. Consequently, camel transportation could explain the more highly diverse fauna of TBPs in camels than that of all other animal hosts in Egypt.

## 8. TBPs in Dogs in Egypt

The majority of the dogs in Egypt are strays. Recently, owning a dog became popular among youth in many urbanized areas. Nonetheless, data on TBPs in dogs from Egypt are scarce. Ten studies that tested 1950 dogs for TBPs were included in the meta-analysis. The most prevalent TBPs in dogs was *Babesia* spp.; 105 out of 924 tested dogs were found to be infected, with a pooled prevalence of 22.8% (CI, 13.0–32.7%). The reports named the species present as *Babesia vogeli* and *Babesia canis* (Table 10). However, the sequenced isolates were completely identical, suggesting that all isolates belonged to the same species, *Babesia canis vogeli*. *Babesia canis* and *Babesia gibsoni* are the two species that are responsible for most canine babesiosis cases worldwide [60]. *Babesia canis* has been further categorized into three subspecies (*B. canis*, *Babesia canis rossi*, and *B. canis vogeli*) [61]. Other tick-borne infections were detected in lower prevalences in dogs from Egypt, such as anaplasmosis (3.5%), ehrlichiosis (5.7%), rickettsioses (1.5%), and borreliosis (0.8%) (see Table 8).

## 9. Tick-Associated Pathogens in Egypt

Egypt has a warm climate, and the temperature often does not drop below 15 °C in the cold months (December–February). Therefore, high tick activity can occur throughout the year. Even in cold months, aggregates of ticks can be noticed on animals. Tick control is an important strategy for combating TBPs that infect animals. In Egypt, a weekly application of acaricides is used by many cattle farms to control ticks, and prolonged incorrect use of the acaricides could result in the development of acaricide-resistant tick populations, reducing the number of effective acaricides in the market and creating a potential future problem for controlling TBPs [62]. Ticks and/or tick pools from 17 studies were combined for estimating the pooled prevalence of various TBPs, and an analysis was conducted in relation to the identified tick genera. In our analysis, ticks belonging to the genus *Boophilus* were moved to the genus *Rhipicephalus*. In the included studies, three genera of Ixodid ticks (*Rhipicephalus*, *Hyalomma*, and *Amblyomma*) were identified and molecularly investigated for their harbored pathogens. Notably, *Theileria* infections were identified in tested ticks from ineligible studies for meta-analysis (Table S6). However, ticks of the genus *Rhipicephalus* (the most frequently tested in 22 datasets) were infected with various *Babesia* (*B. bovis* and *B. bigemina*) and *Anaplasma* (*A. marginale*, *A. platys*, *A. platys-like*, and *A. phagocytophilum*) species. *Borrelia* spp., *Rickettsia* spp., and *C. burnetti* were identified with variable prevalences in the three tested tick genera (Table 11). The pooled prevalence variability and diversity of TBPs in tested ticks was mainly attributed to the use of specific oligonucleotide primers and probes to detect several species of TBPs. Two datasets tested 1248 ticks collected from camels of the genus *Hyalomma* (*H. dromedarii* and *H. rufipes*) and found the Crimean–Congo hemorrhagic fever virus (CCHFV) in 18 (1.4%). Similarly, the same tick species (six pools) that infested camels were found to be positive for CCHFV among the 138 tick pools collected from different animals (Table S6). While the camels that tested positive were imported to Egypt, no reports included this virus in the testing conducted on animals from Egypt. Of note, a study that investigated soft ticks of the genus *Ornithodoros* (*O. savignyi*) detected a high prevalence (66.0%) of *Borrelia burgdorferi*.

Since vertebrate reservoir competence for different pathogens varies widely among species, vector host specificity is critical for understanding the epidemiology of tick-borne infections [63]. Ticks tend to be general global hosts but specialist local hosts [63,64]. Taking into consideration the close interactions of diverse animal species (e.g., sheep and goats), the presence of mixed animal shelters, and the unregulated animal movements in Egypt, the likelihood of a pathogen crossing a species barrier is increased [65,66]. Circulations of some TBPs in Egypt among various ruminants were evident, e.g., *T. annulata*, *B. bigemina*, *B. bovis*, and *A. marginale*. Multiple pathogen co-infections have an impact on tick vector colonization and transmission to vertebrate hosts, and they can be generated either by ticks feeding on the blood of a variety of vertebrate hosts or by co-feeding [67,68].

## 10. Phylogenetic Analysis of *Theileria Annulata*

*Theileria annulata*, the causative agent of bovine tropical theileriosis, causes significant morbidity and mortality in cattle and is a major constraint on the global livestock production [69,70]. Studying the genetic diversity and parasite population structure has become an integral component of epidemiological surveys [71]. A significant number of *T. annulata* isolates from different locations and animals in Egypt were sequenced using the two genetic targets, 18SrRNA and Tams-1. Thus, they were used to study the genetic diversity of this piroplasm all over the country, and the sequencing can be considered a parasite model for the dynamics of TBPs in Egypt.

**Figure 3:**
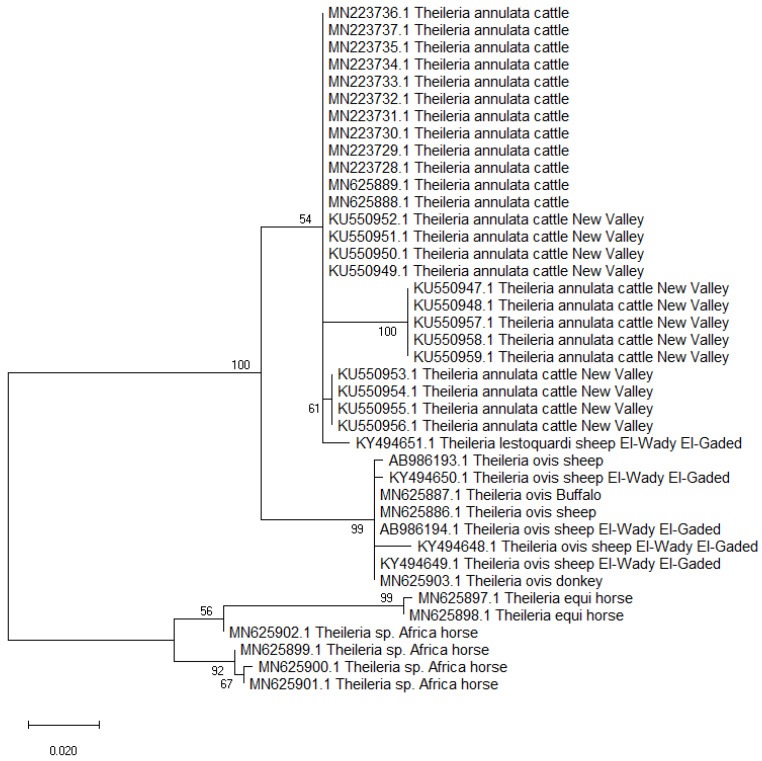
Maximum likelihood tree based on the 18S rRNA gene sequences of *Theileria* sp. isolates from animals in Egypt. The Tamura 3-parameter method was used, which was modeled using a gamma distribution (T92+G). The node numbers represent bootstrap support from 1000 pseudoreplicates.

A total of 40 partial 18S rRNA nucleotide sections (418 nucleotides) were retrieved during the GenBank search, including 25 isolates of *T. annulata* from cattle; 8 *T. ovis* from sheep, buffaloes, and donkeys; 4 *Th.* sp. *Africa* from horses; 2 *T. equi* from horses; and 1 *T. lestoquardi* from sheep. The phylogenetic analysis suggested the suitability of the 18S rRNA in the *Theileria* spp. delimitation; however, lower sequence variabilities were detected within isolates of the same species. The *Theileria annulata* isolates from Egypt were divided into three subclades in a clade that also included *T. lestoquardi* from sheep, suggesting that both species have a common ancestor [72]. The isolates of *T. ovis* from sheep, buffaloes, and donkeys were clustered together in a separate clade but in the same branch that included the *T. annulata* clade. Meanwhile, the isolates of *Th.* sp. *Africa* and *T. equi* from horses were genetically related and arranged in the second branch (Figure 3). The 18S rRNA gene had varying levels of genetic variations among different isolates globally, but Tams-1 gene was previously reported to be highly polymorphic [69,71].

**Figure 4:**
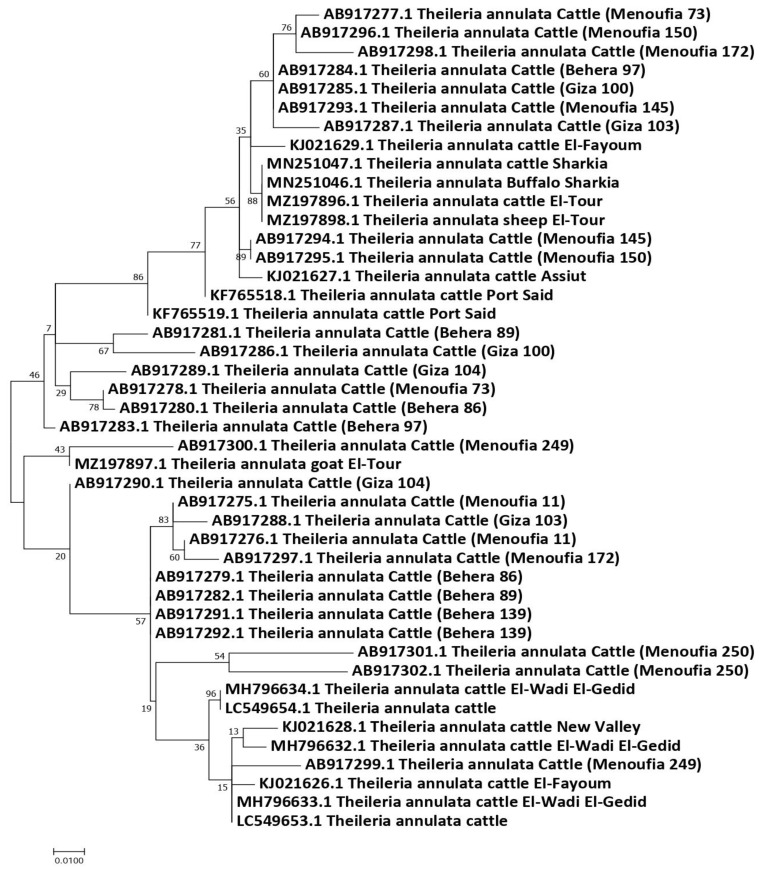
Maximum likelihood tree based on the Tams-1 gene sequences of *T. annulata* from animals in Egypt. The Hasegawa–Kishino–Yano (HKY) model was used. The node numbers represent values with bootstrap support from 1000 pseudoreplicates.

**Figure 5:**
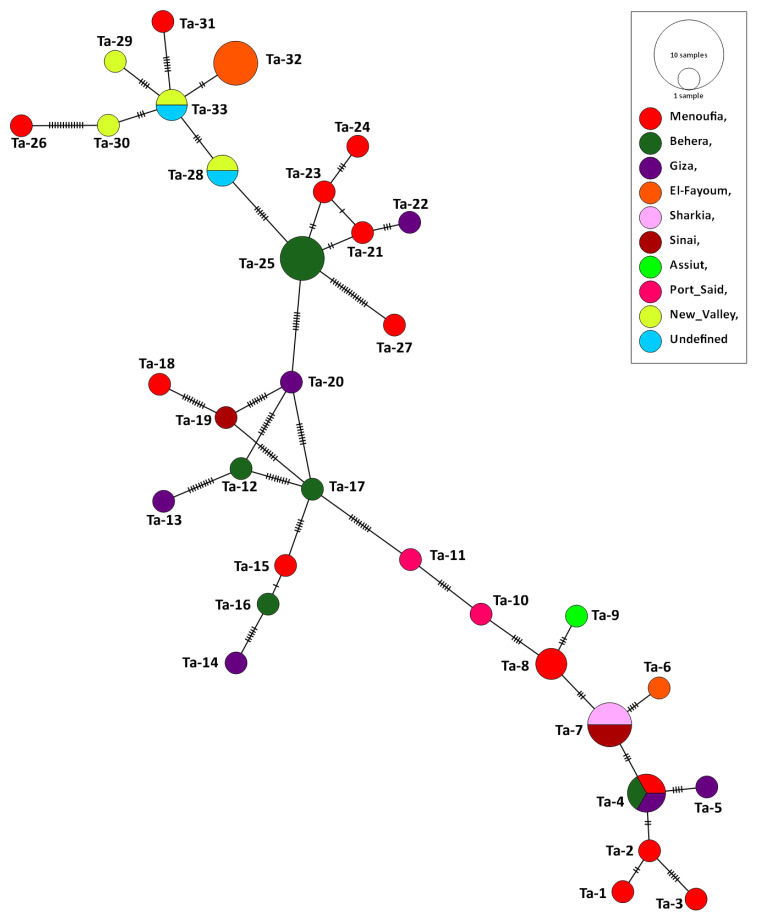
Haplotype network established based upon the Tams-1 nucleotide sections of *T. annulata* isolates from Egypt. The network describes the distribution of the revealed haplotypes in relation to the governorates of origin, indicated by different colors. The circle sizes are consistent with the haplotype frequency. The number of mutations distinguishing the haplotypes is shown by the hatch marks.

Forty-four partial Tams-1 sequences for *T. annulata* were used for the genetic analysis. Those isolates came from animals in Egypt: cattle (*n* = 41), sheep (1), buffalo (1), and goat (1). After aligning the revealed sequences, the sequences length of 277 nucleotides were revealed, and the total number of sites that were used for the analysis was 273, excluding sites with gaps/missing data (*n* = 4). The genetic analysis confirmed the highly variable nature of Tams-1; 33 haplotypes were detected out of the 44 studied isolates, giving rise to a very high haplotype diversity (0.981). In total, 63 polymorphic sites were detected, with a nucleotide diversity of 0.06331, and the total number of mutations (Eta) was 73. The average number of nucleotide differences (k) was 17.28436, and the Tajima’s D neutrality index was 0.10712, which was statistically insignificant. The revealed haplotypes were named Ta-1–Ta-33 (Table S7). Out of the 33 detected haplotypes, 27 were singleton, and 6 had a shared status. The haplotype Ta-7 included *T. annulata* isolates from cattle (*n* = 2), buffalo (1), and sheep (1) in two very separate regions (Sharkia and Sinai). In addition, 3 *T. annulata* isolates from cattle in three different governorates (Giza, Behera, and Menoufia) shared the haplotype Ta-4, suggesting an absence of haplotype distribution in relation to the host species or geographic location. However, the goat isolate formed a singleton haplotype (Ta-19) (Figure 4 and Figure 5).

## 11. Conclusions

Our study provides the first meta-analysis of published molecular data on TBPs in Egypt. Nonetheless, several important aspects were highlighted for the status of TBPs, which have serious health and economic implications on the animal industries in Egypt, particularly babesiosis, theileriosis, and anaplasmosis. There is evidence of high species diversity of the TBPs infecting animals from Egypt, which suggests endemicity and complex transmissions. Animals from Egypt and their infesting ticks were found to harbor many zoonotic and/or potentially zoonotic pathogens, such as *A. phagocytophilum* (anaplasmosis), *B. microti* and *B. divergens* (babesiosis), *Borrelia burgdorferi* (Lyme disease), *Coxiella burnetii* (Q fever), rickettsiosis, CCHFV (Crimean–Congo hemorrhagic fever), and Ehrlichia spp. (ehrlichiosis), which can be transmitted to their accompanying farmers. Ticks that infest animals and their associated pathogens displayed high species diversity, underlining the high infection risk to animals as well as constituting a reservoir for a wide range of zoonotic TBPs. Adequate control measures against TTBPs should be applied to prevent their circulation among animals in the country.

## Figures and Tables

**Table 1:** Keywords used for searching different databases.

Pathogens and Diseases	Animals	Methods	Country	Databases
tick-borne diseases *Babesia* babesiosis *Theileria* theileriosis *Anaplasma* anaplasmosis *Coxiella burnetii* Q fever *Ehrlichia* ehrlichiosis *Rickettsia* rickettsioses *Borrelia* borreliosis CCHF Crimean–Congo haemorrhagic fever	cattle buffaloes sheep goats camels equines horses donkeys dogs	Molecular PCR	Egypt	PubMed Scopus ScienceDirec tEgyptian Knowledge Bank Google Scholar

**Table 2:** Partial nucleotide sequences of *T. annulata* Tams-1 and 18srRNA isolates from ruminant animals in Egypt used for clustering analysis.

Host	Species	Marker	GenBank Accession Number	Length (bp)	Reference
Cattle	*T. annulata*	*Tams-1*	KF765518 and KF765519	276	[20]
Cattle	*T. annulata*	*Tams-1*	KJ021626–KJ021629	777–789	[21]
Cattle	*T. annulata*	*Tams-1*	AB917275–AB917302	771–783	[22]
Cattle	*T. annulata*	*Tams-1*	AB917275, AB917298, AB917299, AB917300, and AB917302	771–783	[23]
Cattle	*T. annulata*	*18S rRNA*	KU550947–KU550959	414–437	[24]
Cattle	*T. annulata*	*Tams1*	MN251047	702	[25]
Cattle	*T. annulata*	*Tams1*	MH796632–MH796634	622	[26]
Cattle	*T. annulata*	*18S rRNA*	MN625888 and MN625889	910, 912	[27]
Cattle	*T. annulata*	*18S rRNA*	MN223728–MN223737	535–631	[28]
Cattle	*T. annulata*	*Tams-1*	MZ197896	636	[29]
Cattle	*T. annulata*	*Tams-1*	LC549653 and LC549654	620	[30]
Buffalo	*T. annulata*	*Tams1*	MN251046	702	[25]
Buffalo	*T. ovis*	*18S rRNA*	MN625887	919	[27]
Sheep	*T. ovis*	*18S rRNA*	MN625886	919	[27]
Sheep	*T. ovis* *T. lestoquardi*	*18S rRNA*	AB986193 and AB986194 KY494648–KY494650 KY494651	434 391–395 416	[31]
Sheep	*T. annulata*	*Tams1*	MZ197898	636	[29]
Goats	*T. annulata*	*Tams1*	MZ197897	636	[29]
Horses	*T. equi*	*18S rRNA*	MN625897 and MN625898	924	[32]
Horses	*T. sp. Africa*	*18S rRNA*	MN625899–MN625902	920–925	[32]
Donkeys	*T. ovis*	*18S rRNA*	MN625903	919	[32]

**Table 3:** Pooled prevalence of TBPs detected in cattle from Egypt and prevalence variation in relation to the species detected.

Parameter	No. Data Sets	No. Tested	No. Positive	Pooled Estimate % Based on 95% CI	Heterogeneity *I^2^*%
*Babesia*	14	3203	525	16.0 (10.9–21.0)	95.6
*B. bigemina*	12	2855	328	10.1 (6.3–13.8)	96.7
*B. bovis*	9	2129	177	9.5 (6.0–13.0)	90.6
*B. ovis*	1	164	12	7.3 (3.3–11.3)	N/A
*B. occultans*	1	309	1	0.3 (−0.3–1.0)	N/A
*Babesia spp.*	1	164	13	7.9 (3.8–12.1)	N/A
*Theileria*	16	4620	1324	36.0 (23.4–48.7)	99.5
*T. annulata*	13	3865	1200	30.8 (18.9–42.7)	98.9
*T. orientalis*	3	1378	50	3.0 (0.3–5.6)	94.9
*Anaplasma*	9	1745	510	43.9 (4.8–83.1)	99.9
*A. marginale*	7	1601	328	21.2 (4.6–37.7)	98.7
*A. centrale*	2	128	2	1.4 (0.6–3.4)	0.0
*A. platys-like*	2	180	16	8.3 (2.1–18.8)	85.3
*A. platy*	1	309	26	8.4 (5.3–11.5)	N/A
*A. phagocytophilum*	1	40	6	15.0 (3.9–26.1)	N/A
*A. ovis*	1	88	3	3.4 (0.4–7.2)	N/A
*Anaplasma spp.*	1	40	10	25.0 (11.6–38.4)	N/A
*Bartonella*	2	200	6	2.6 (−2.1–7.2)	77.6
*Borrelia*	3	225	8	2.9 (1.1–7.0)	62.6
*Borrelia theileri*	2	200	1	0.7 (−0.5–1.9)	0.0
*Borrelia burgdorferi*	2	96	25	23.7 (10.5–36.9)	54.9
*C. burnetti*	3	152	12	7.2 (1.8–16.2)	88.1
*Rickettsia*	3	240	6	1.1 (−1.2–3.3)	69.6

**Table 4:** Pooled prevalence of TBPs detected in buffaloes from Egypt and prevalence variation in relation to the species detected.

Parameter	No. Data Sets	No. Tested	No. Positive	Pooled Estimate % Based on 95% CI	Heterogeneity *I^2^*%
*Babesia*	5	398	21	3.6 (0.6–6.6)	69.2
*B. bigemina*	4	408	12	1.9 (0.1–3.9)	65.7
*B. bovis*	4	368	9	2.1 (0.6–3.5)	0.0
*Theileria*	4	247	4	1.0 (0.2–2.2)	0.0
*T. annulata*	2	107	12	24.4 (−23.4–72.2)	95.2
*T. orientalis*	1	50	1	2.0 (−1.9–5.9)	N/A
*T. ovis*	1	26	2	7.7 (−2.6–17.9)	N/A
*Anaplasma*	4	347	132	26.9 (7.3–61.1)	99.1
*A. marginale*	3	321	141	37.5 (10.4–85.8)	99.4
*A. platy-like*	1	26	2	7.7 (2.6–17.9)	N/A
*A. platys*	1	85	4	4.7 (0.9–9.2)	N/A
*Bartonella*	2	52	3	5.0 (−3.9–13.9)	51

**Table 5:** Pooled prevalence of TBPs detected in sheep from Egypt and prevalence variation in relation to the species detected.

Parameter	No. Data Sets	No. Tested	No. Positive	Pooled Estimate % Based on 95% CI	Heterogeneity *I^2^*%
*Babesia*	3	279	14	3.8 (0.4–8.1)	79.1
*B. bovis*	3	279	12	2.7 (0.7–6.2)	79.4
*B. bigemina*	1	105	2	1.9 (0.7–4.5)	N/A
*B. ovis*	1	66	6	9.1 (2.2–16.0)	N/A
*Theileria*	3	281	33	11 (2.3–19.7)	83.2
*T. annulata*	1	108	22	20.4 (12.8–28.0)	N/A
*T. ovis*	2	173	10	5.3 (1.8–8.8)	5.8
*T. lestoquardi*	1	115	1	0.9 (−0.8–2.6)	N/A
*Anaplasma*	4	599	106	16.1 (6.6–23.5)	89.4
*A. marginale*	2	178	4	2.0 (0.0–4.1)	0.0
*A. ovis*	3	536	61	7.1 (1.0–15.1)	93.6
*A. phagocytophilum*	1	120	12	10.0 (4.6–15.4)	N/A
*A. platys*	1	120	2	1.7 (0.6–4.0)	N/A
*A. platys-like*	1	58	1	1.7 (−1.6–5.1)	N/A
*Anaplasma* spp.	1	120	20	16.7 (10.0–23.3)	N/A
*Bartonella*	2	96	3	3.1 (−3.3–9.6)	58.6
*Borrelia theileri*	1	58	2	3.4 (1.2–8.1)	NA
*Rickettsia*	2	168	30	13.7 (−12.1–39.6)	97.2
*C. burnetti*	6	369	94	45.3 (9.5–81.2)	99.5

**Table 6:** Pooled prevalence of TBPs detected in goats from Egypt and prevalence variation in relation to the species detected.

Parameter	No. Data Sets	No. Tested	No. Positive	Pooled Estimate % Based on 95% CI	Heterogeneity *I^2^*%
*Babesia bovis*	1	48	8	16.7 (6.1–27.2)	N/A
*Theileria Annulata*	1	48	24	50.0 (35.9–64.1)	N/A
*Bartonella*	2	61	1	2.0 (−1.5–5.5)	0.0
*C. burnetti*	5	187	46	29.4 (6.2–52.7)	97.1

**Table 7:** Pooled prevalence of TBPs detected in horses from Egypt and prevalence variation in relation to the species detected.

Parameter	No. Data Sets	No. Tested	No. Positive	Pooled Estimate % Based on 95% CI	Heterogeneity *I^2^*%
*Babesia*	2 (*B. caballi*)	167	18	9.8 (−7.8–27.5)	94.1
*Theileria*	6	847	229	34.1 (12.9–55.3)	98.7
*T. equi*	6	847	181	25.4 (7.3–43.5)	98.5
*T. haneyi*	1	79	42	53.2 (42.2–64.2)	N/A
*T. sp. Africa*	1	320	9	2.8 (1.0–4.6)	N/A
*Bartonella*	2	328	1	0.8 (−4.6–6.1)	10.2

**Table 8:** Pooled prevalence of TBPs detected in donkeys from Egypt and prevalence variation in relation to the species detected.

Parameter	No. Data Sets	No. Tested	No. Positive	Pooled Estimate % Based on 95% CI	Heterogeneity *I^2^*%
*Babesia*	2 (*B. caballi*)	127	8	7.2 (−7.2–21.5)	88.1
*Theileria*	7	524	158	30.6 (14.0–47.2)	95.2
*T. equi*	6	510	145	3.6 (13.4–57.7)	95.9
*T. haneyi*	1	76	29	38.2 (27.2–49.1)	N/A
*T. ovis*	1	15	2	13.3 (−3.9–30.5)	N/A
*Anaplasma*	1	15	4	26.7 (4.3–49.2)	N/A
*A. marginale*	1	15	2	13.3 (−3.9–30.5)	N/A
*A. ovis*	1	15	2	13.3 (−3.9–30.5)	N/A
*Bartonella*	2	37	2	5.1 (−1.8–12.1)	0.0

**Table 9:** Pooled prevalence of TBPs in dromedary camels from Egypt and prevalence variation in relation to the species detected.

Parameter	No. Data Sets	No. Tested	No. Positive	Pooled Estimate % Based on 95% CI	Heterogeneity *I^2^*%
*Babesia*	3	615	82	11.0 (0.7–21.2)	95.0
*B. bovis*	2	473	40	6.8 (1.1–14.7)	92.5
*B. bigemina*	1	331	25	7.6 (4.7–10.4)	N/A
*B. microti*	1	142	17	12.0 (6.6–17.3)	N/A
*Theileria*	2	361	259	71.8 (67.1–76.4)	0.0
*T. annulata*	1	30	18	60.0 (42.5–77.5)	N/A
*T. camelensis*	1	331	238	71.9 (67.1–76.7)	N/A
*Theileria* spp.	1	30	3	10.0 (0.7–20.7)	N/A
*Anaplasma*	4	690	327	40.5 (6.4–74.6)	99.2
*A. marginale*	3	590	234	25.7 (13.8–65.1)	99.7
*A. centrale*	2	441	178	28.7 (16.9–74.3)	99.4
*A. phagocytophilum*	1	110	20	18.2 (11.0–25.4)	N/A
*A. ovis*	1	110	6	5.5 (1.2–9.7)	N/A
*A. bovis*	1	110	5	4.5 (0.7–8.4)	N/A
*A. platys*	2	259	6	2.2 (−1.5–5.8)	70.4
*A. platys-like*	1	149	8	5.4 (1.7–9.0)	N/A
*Ca. An. cameli*	1	100	29	29.0 (20.1–37.9)	N/A
*Anaplasma* spp.	1	110	13	11.8 (5.8–17.9)	N/A
*C. burnetti*	3	374	71	20.8 (3.8–45.3)	98.3
*Rickettsia*	3	330	91	31.9 (8.4–72.2)	98.9

**Table 10:** TBPs detected in dogs from Egypt and prevalence variation in relation to the species detected.

Parameter	No. Data Sets	No. Tested	No. Positive	Pooled Estimate % Based on 95% CI	Heterogeneity *I^2^*%
*Babesia*	6	924	105	22.8 (13.0–32.7)	98.9
*B. vogeli*	3	592	90	16.3 (1.5–31.1)	95.8
*B. canis*	1	203	1	0.5 (−0.5–1.5)	N/A
*Anaplasma*	3	819	40	3.5 (0.1–6.9)	87.5
*A. platys*	2	703	39	5.0 (2.1–7.9)	67.4
*Borrelia*	4	445	8	0.8 (−0.7–2.4)	64.2
*B. burgdorferi*	2	126	7	10.5 (10.9–31.9)	85.8
*Ehrlichia*	2	516	41	5.7 (−0.2–13.6)	94.3
*Rickettsia*	1	203	3	1.5 (−0.2–3.1)	N/A

**Table 11:** Pooled prevalence of TBPs detected in ticks collected from animals in Egypt and prevalence variation in relation to the species detected.

Parameter	No. Data Sets	No. Tested	No. Positive	Pooled Estimate % Based on 95% CI	Heterogeneity *I^2^*%
Rhipicephalus	22	5053	249	10.1 (7.3–13.0)	95.7
*Babesia* *	3	372	123	40.6 (7.1–88.2)	99.3
*B. bovis*	1	100	55	55.0 (45.2–64.8)	N/A
*B. bigemina*	2	272	68	33.4 (−30.1–97.0)	99.4
*Anaplasma* *	4	679	59	4.5 (0.8–9.8)	93.2
*A. marginale*	1	61	1	1.6 (−1.5–4.8)	N/A
*A. platys*	1	156	2	1.3 (−0.5–3.0)	N/A
*A. platys-like*	1	61	1	1.6 (−1.5–4.8)	N/A
*A. phagocytophilum*	1	401	55	13.7 (10.3–17.1)	N/A
*Borrelia* *	7	439	28	5.4 (1.6–9.2)	69.3
*Borrelia* spp.	1	61	2	3.3 (−1.2–7.7)	N/A
*B. theileri*	2	233	12	4.9 (2.1–7.6)	0.0
*B. burgdorferi*	4	145	14	11.4 (0.5–22.4)	83.5
*C. burnetti*	1	28	1	3.6 (−3.3–10.4)	N/A
*Ehrlichia*	2	217	10	5.9 (−3.3–15.1)	80.4
*Rickettsia*	5	3342	48	10.9 (3.9–17.9)	93.6
Ornithodoros	1 (*B. burgodorferi*)	47	31	66.0 (52.4–79.5)	N/A
Hyalomma	20	3700	328	21.0 (16.1–26.0)	97.3
*B. burgdorferi*	3	66	22	31.9 (14.9–48.9)	58
*C. burnetti*	2	436	27	6.2 (3.9–8.4)	0.0
*CCHFV*	2	1248	18	1.4 (0.8–2.1)	0.0
*Rickettsia*	13	1950	261	27.0 (14.5–39.5)	97.4
Amblyomma	6	224	46	16.7 (6.9–26.4)	77.6
*B. burgdorferi*	1	14	4	28.6 (4.9–52.2)	N/A
*C. burnetti*	2	37	2	5.4 (−1.9–12.7)	0.0
*Rickettsia*	3	173	40	22.2 (7.2–37.3)	84.5

* Mixed species infections within the same genus were not excluded.

## Data Availability

Not applicable.

## Supplementary Materials

The following supporting information can be downloaded at: https://www.mdpi.com/article/10.3390/pathogens11080912/s1, Table S1: Study characteristics of tick-borne pathogens molecular surveys in cattle and buffaloes from Egypt.; Table S2: Study characteristics of tick-borne pathogens molecular surveys in sheep and goats from Egypt.; Table S3: Study characteristics of tick-borne pathogens molecular surveys in equines from Egypt.; Table S4: Study characteristics of tick-borne pathogens molecular surveys in dromedary camels (Camelus dromedarius) from Egypt; Table S5: Study characteristics of tick-borne pathogens molecular surveys in dogs from Egypt.; Table S6: Study characteristics of molecular surveys of pathogens in ticks infesting livestock animals from Egypt.; Table S7: Haplotypes of T. annulata Tams-1 isolates from ruminant animals in Egypt.
