# Supplementary material for: Molecular Epidemiology and Species Diversity of Tick-Borne Pathogens of Animals in Egypt: A Systematic Review and Meta-Analysis
Source: Pathogens. 2022 Aug 14;11(8):912. doi: 10.3390/pathogens11080912 (PMC9416077; doi:10.3390/pathogens11080912)
Supplement: Supplementary file 1 [file pathogens-11-00912-s001.zip › pathogens-1827594-supplementary.pdf]

Supplementary Table S1: Study characteristics of tick-borne pathogens molecular surveys in cattle and buffaloes from Egypt.

Reference	Region	Host	Study Year	Sample size	No. positive (%)	Marker	Species	Molecular tools	Sequenced isolates	Accession numbers
Mahmmod et al., 2010¥	Sharkeya, Dekhlia, Ismalia	Bovine	NS	30#	21 (70)	NS	*Th. annulata*	cPCR	-	-
Abd Ellah and AL-Hosary, 2011	Assuit	cattle	NS	28	27 (96.42)	Tams-1	*Th. annulata*	cPCR	-	-
Nayel et al., 2012	Menofia	cattle	NS	158	20 (12.66)<br>38 (24.05)	18S rRNA	*Babesia sp.*<br>*Theileria sp.*	cPCR	-	-
Ibrahim et al., 2013	Beheira and Faiyum	cattle	2011	151	8 (5.30)<br>6 (3.97)<br>0 (0)	RAP-1a<br>SBP-4	*B. bigemina*<br>*B. bovis*<br>Mixed sp.	nPCR	3<br>2	KF192810-KF192812<br>KF192806-KF192808
		buffaloes		96	10 (10.42)<br>4 (4.17)<br>0 (0)	RAP-1a<br>SBP-4	*B. bigemina*<br>*B. bovis*<br>Mixed sp.		2<br>1	KF192809<br>KF192805
Abdel Aziz et al., 2014	Sharkeya	cattle	NS	296	117 (39.5)<br>44 (14.8)<br>73 (24.7)	 RRA<br>RAP-1	*Babesia sp*<br>*B. bovis*<br>*B. bigemina*	nPCR	-	-
Elhelw et al., 2014	NS	cattle	2008-2009	25	4 (16)	OspA	*B. burgdorferi*	cPCR	1	KC522030
Ghoneim and El-Fayomy, 2014	Port Said	cattle	NS	64	44 (68.8)	Tams-1	*Th. annulata*	cPCR	2	KF765518 and KF765519
AL-Hosary et al., 2015	Upper Egypt	cattle	2010-2013	210	97 (46.19)	Tams-1	*Th. annulata*	cPCR	4	KJ021626- KJ021629
El-Ashker et al., 2015	Dakahlia	cattle	2012-2013	164	13<br>12<br>2<br>35	18S rRNA<br><br><br>16S rRNA	*Babesia sp.*<br>*B. bovis*<br>*B. bigemina*<br>*A. marginale*	cPCR<br>DNA array<br>cPCR	NS	Sequencing performed but, no GeneBank submitted accession numbers
Elsify et al., 2015	Menoufia, Behera, Giza, and Sohag	cattle	2013	439	14 (3.18)<br>35 (7.97)<br>4 (0.9)<br>42 (9.56)<br>3 (0.68)	RAP-1<br>AMA-1<br><br>Tams-1<br>MPSP	*B. bovis*<br>*B. bigemina,*<br>Mixed Babesia<br>*T. annulata,*<br>*T. orientalis*	nPCR<br>cPCR<br><br>cPCR<br>cPCR	11<br>10<br><br>14<br>3	AB917246-AB917257<br>AB917263-AB917274<br><br>AB917275-AB917302<br>AB917303 and AB917305

					4 (0.9)		Mixed Theleiria			
		buffaloes		50	1 (2.00)	RAP-1	*B. bovis*	nPCR	1	AB917258
					1 (2.00)	MPSP	*T. orientalis*	cPCR	1	AB917306
Mahmoud et al.,	Kafr El-	cattle	NS	253	38 (15.0)	rra	*B. bovis*	nPCR	1	KM213000
2015	Sheikh, El-				82 (32.4)	rap-1c	*B. bigemina*		1	KM212998
	Beheira,				22 (8.7)		Mixed 2 sp.			
	Cairo	buffaloes		81	3 (3.7)	rra	*B. bovis*			
					0 (0.0)	rap-1c	*B. bigemina*			
Elhaig et al.,	Ismailia,	cattle	2013-	500	55 (11)	18S rRNA	*B. bigemina*	cPCR	1	KM076937
2016	Sharkia and		2014							
	Qalubiya									
AL-Hosary,	Assuit	cattle	NS	76	13 (17.11)	18S rRNA	*B. bovis*	cPCR	3	KM455548, KM455549 and
2017					20 (26.3)			nPCR		KM455550
					20 (26.3)			PCR-RLB		
Abdel-Moein	Giza	cattle	NS	26	0	IS1111	*C. burnetii*	nPCR	-	-
and Hamza,		buffaloes		26	0					
2017										
ELHariri et al.,	Giza,	buffaloes	NS	150	104 (69.3)	msp1α	*A. marginale*	cPCR	-	-
2017	Qalyoubia,									
	El-Wadi									
	El-Gadeed,									
	Menofia									
Rizk et al., 2017	Menoufia,	cattle	2013	439	49 (11.16)		*Babesia sp*			
	Behera,					RAP-1	*B. bovis*	nPCR	5	AB917246, AB917251, AB917253, AB917255, and AB917257
	Giza, and					AMA-1	*B. bigemina*	cPCR	2	AB917263 and AB917274
	Sohag				45 (10.25)		*Theileria sp.*			
						Tams-1	*T. annulata*	cPCR	5	AB917275, AB917298, AB917299, AB917300and AB917302
						MPSP	*T. orientalis*	cPCR	2	AB917303 and AB917304
AL-Hosary et	EL-Wady	cattle	2015-	1068	679 (63.6)	Tams-1,	*Th. annulata*	cPCR	13	
al., 2018	EL-Geded		2016		------	18Ss rRNA		nPCR		KU550947- KU550959
Abdel-Baky and	Variable	cattle	2014-	40	23 (17.83)	16S rRNA	***Anaplasmataceae***	cPCR	**-**	Sequencing performed but, no
Allam, 2018			2016		6	msp4 and	*A. phagocytophilum*			GeneBank submitted
					4	hsp60	*A. marginale*			accession numbers
					2		*A. bovis*			
					1		*A. centrale*			
					10		*Anaplasma spp.*			

Allam et al., 2018	Variable	cattle	2014-2016	40	6 (5.88)	16S rDNA, OmpA and gltA	*Rickettsiae*	cPCR	-	Sequencing performed but, no GeneBank submitted accession numbers
El-Dakhly et al., 2018	El-Wadi El-Gadid	cattle	NS	376	43 (11.44)	Cytb1	*T. annulata*	cPCR	-	-
Anter et al., 2019¥	Sharkia	cattle	2018	30#	NS	Tams1	*T. annulata*	cPCR	1	MN251047
		buffaloes		29#	NS				1	MN251046
AL-Hosary et al., 2020®	EL-Minia and Assiut, ELFayoum, and New Valley	cattle	2018	309	211 (68.3)	msp1β	*A. marginale*	qPCR		MN227687, MN227689-MN227692
					155 (50.2)	groEL	*A. marginale*	RLB		
					49 (15.9)	18S rRNA	*T. annulata*	RLB		
					18 (5.8)	18S rRNA	*B. bovis*	RLB		MN223723-MN223737
					2 (0.7)	18S rRNA	*B. bigemina*	RLB		
					1 (0.3%)	18S rRNA	*B. occultans*	RLB		
					26 (8.4)	groEL	*A. platys*	RLB		MN227676-MN227679 MN227675
		buffaloes		85	25 (29.4)	msp1β	*A. marginale*	qPCR		
					36 (42.4)	groEL	*A. marginale*	RLB		MN202017-MN202023 and MN227688
					1 (1.18)	18S rRNA	*T. annulata*	RLB		
					2 (2.35)	18S rRNA	*B. bigemina*	RLB		
					4 (4.71)	16S rRNA	*A. platys*	RLB		
Abbass et al., 2020	Assiut	cattle	2016-2017	38*	12 (31.6)	IS1111	*C. burnetii*	RT-qPCR	-	-
Abdel-Baky et al., 2020¥	NS	cattle	2014-2016	40	22.22%	OspA and flaB	*B. burgdorferi*	nPCR	-	-
El-Dakhly et al., 2020	Beni-Suef, El-Fayoum and El-Wadi El-Gadid	cattle	2015-2018	150	33 (22)	Tams1	*T. annulata*	cPCR	3	MH796632- MH796634
					29 (19.33)	SSrRNA	*B. bigemina*	cPCR	3	MH796638-MH796640
					16 (10.6)	msp1b	*A. marginale*	cPCR	3	MH796635-MH796637
Nasreldin et al., 2020	New Valley	cattle	2017-2018	14#	12	msp1b	*A. marginale*	cPCR	-	-
					14	VESA–1a	*B. bovis*	cPCR		
Tumwebaze et al., 2020	Menoufia	cattle	2017	92	5 (5.4)	SBP4	*B. bovis*	nPCR	4	MN870658-MN870661
					6 (6.5)	Rap1a	*B. bigemina*	nPCR	3	MN870655-MN870657
					14 (15.2)	groEL	*A. marginale*	nPCR	4	MN870643-MN870646
					13 (14.1)	16S rRNA	*Anaplasma sp. (A. platys-like)*	nPCR	6	MN861059-MN861064
					9 (9.8)		Mixed *Anaplasma*			

					0 (0)	18S rRNA	*Theileria sp.*	nPCR		
		buffaloes		86	1(1.2)	SBP4	*B. bovis*	nPCR		
					0 (0.0)	Rap1a	*B. bigemina*	nPCR		
					1(1.2)	groEL	*A. marginale*	nPCR	1	MN870645
					0 (0)	18S rRNA	*Theileria sp.*	nPCR		
Parvizi et al., 2020a	variable	cattle	2015-2016	758	40 (5.3)	-	*Anaplasma/Ehrlichia*	qPCR	-	-
						16S rRNA	*A. marginale*			
Yousef et al., 2020	Sharkia	cattle	2019	25#	25	Tams1	*T. annulata*	cPCR	-	-
Abdullah et al., 2021a	variable	cattle	2019-2020	112	6 (5.36)	ITS	*Bartonella sp.*	cPCR	1	MW596416
					1 (0.89)	16S rRNA	*Borrelia theileri*		1	MW562684
					0 (0)	gltA	*Rickettsiae*			
		buffaloes		26	3 (11.54)	ITS	*Bartonella sp.*		1	MW596417
					0 (0.0)	16S rRNA	*Borrelia theileri*			
					0 (0)	gltA	*Rickettsiae*			
Abdullah et al., 2021b	Beni-Suef, Qalyubia, El-Wady El-Geded, Qena, Beheira	cattle	2016-2018	88	15 (17)	5.8S rRNA	*Piroplasmida*	qPCR		
					14 (15.9)	18S rRNA	*T. annulata*	nPCR		MN625888 and MN625889
					1 (1.1)	18S rRNA	*Ba. bigemina*	nPCR		MN625890
					25 (28.4)	23S rRNA	*Anaplasmataceae*	qPCR		
					18 (20.4)	Ana-rpoB	*An. marginale*	cPCR		MN625935, MN626393, MN624134
					1 (1.1)	Ana-rpoB	*An. centrale*	cPCR		MN626394
					3 (3.4)	Ana-rpoB	*An. ovis*	cPCR		MN625933, MN624133
					3 (3.4)	Ana-rpoB	*An. platys-like*	cPCR		MN626397, MN626400, MN624137
					3 (3.4)	16S rRNA	*Borrelia sp.*	qPCR		MN621893
					0	16S RNA	*Bo. theileri*	cPCR		
					0	gltA	*Rickettsia sp.*	qPCR		
					0	IS1111	*C. burnettii*	qPCR		
					0	ITS3	*Bartonella sp*	qPCR		
		buffaloes		26	2 (7.7)	5.8S rRNA	*Piroplasmida*	qPCR		
					2 (7.7)	18S rRNA	*T. ovis*	cPCR		MN625887
					2 (7.7)	23S rRNA	*Anaplasmataceae*	qPCR		
					2 (7.7)	Ana-rpoB	*An. platys-like*	cPCR		MN626399, MN624139
					0	gltA	*Rickettsia sp.*	qPCR		
					0	IS1111	*C. burnettii*	qPCR		
					0	ITS3	*Bartonella sp*	qPCR		
AL-Hosary et al. 2021b¥	Faiyum, Assiut and Kharja	cattle	2018	41	39	18S rRNA	*T. annulata*	RLB, cPCR		MN223728:MN223732
					38	16S rRNA	*An. marginale*			MN370071:MN370075
					2	18S rRNA	*B. bovis*			

					2<br>1	18S rRNA<br>18S rRNA	*B. bigemina*<br>*B. occultans*			MN227677 and MN227679
El Damaty et al., 2021¥	El-Sharkia	cattle<br>buffaloes	2019-2020	48<br>22	24<br>9	Tams-1,<br>18s rRNA	*T. annulata*	cPCR	-	-
Selim et al., 2021a	Kafr El-Sheikh, Menofia, and Al-Gharbia	cattle	2020	130*	130 (100)<br>0<br>0	MSP4	*A. marginale*<br>*A. centrale*<br>*A. bovis*	cPCR	2	MZ695054 and MZ695055
Barghash, 2022	Sinai	cattle	2020-2021	74	18 (24.32)<br>0 (0)<br>30 (40.54)	BbSBP-4<br>RAP-1a<br>tams1	*B. bovis*<br>*B. bigemina*<br>*T. annulata*	cPCR	1<br><br>1	MZ197893<br><br>MZ197896
Selim et al., 2022b	Gharbia, Beheira, Kafr ElSheikh, Menofia	cattle	2020	500	44 (8.8)	MPSP	*T. orientalis*	cPCR	3	LC661355-LC661357
Selim et al., 2022c	Alexandria, Beheira, Kafr El Sheikh, Qalyubia, Menofia	cattle	2019-2020	570	94 (16.49)	Tams-1	*T. annulata*	cPCR	2	LC549653 and LC549654

*Seropositive samples

#Positive by microscopy

®Infections were detected using qPCR for A. marginale and PCR-RLB followed by sequencing for all detected pathogens as well as conventional and semi-nested PCRs/sequencing for confirmations of genotyping results.

¥ Studies not included in Meta-analysis.

Abbreviations: **AMA-1;** Apical Membrane Antigen-1, **BbSBP-4**; B. bovis Rhoptry Associated Protein-4, **cytb**; Cytochrome b, **gltA**; Citrate synthase, **groEL**; 'heatshock operon', **htp*B***; Heat shock protein antigenic polypeptide, **IS1111**; C. burnetii transposon-like sequence, **ompA**; Outer membrane protein A, **MPSP;** Major Piroplasm Surface Protein gene, **msp1b;** major surface protein-1b gene, **ospA**; Outer surface protein A. gene, **RAP-1;** Rhoptry Associated Protein-1, **RRA;** Rhoptry associated protein related antigen, **SSrRNA;** small subunit ribosomal RNA gene, **Tams-1**; Theileria annulata 30 KDa major merozoite surface antigen-1 gene

Supplementary Table S2: Study characteristics of tick-borne pathogens molecular surveys in sheep and goats from Egypt.

Reference	Region	Host	Study Year	Sample size	No. positive (%)	Marker	Species	Molecular tools	Sequenced isolates	Accession numbers
Elsify et al., 2015	Menoufia, Behera, Giza	sheep	2013	105	1 (0.95)<br>2 (1.90)	RAP-1<br>AMA-1	*B. bovis*<br>*B. bigemina*	nPCR<br>cPCR	1<br>2	AB917259<br>AB917260-AB917262
Khalifa et al., 2016	Qaluobia	sheep<br>goats	2014-2015	23*<br>27*	21 (91.3)<br>23 (85.2)	IS1111	*C. burnetii*	trans-PCR	-	-
Abdel-Moein and Hamza, 2017	Giza	goats<br>sheep	NS	29#<br>27	1 (3.4)<br>0	IS1111	*C. burnetii*	nPCR	1	KU977532
Abdel-Baky and Allam, 2018	Variable	sheep	2014-2016	120	41 (31.78)<br>12<br>2<br>5<br>2<br>20	16S rRNA msp4 and hsp60	***Anaplasmataceae***<br>*A. phagocytophilum*<br>*A. marginale*<br>*A. ovis*<br>*A. platys*<br>*Anaplasma spp.*	cPCR	**-**	Sequencing performed but, no GeneBank submitted accession numbers
Allam et al., 2018	Variable	sheep	2014-2016	110	30 (29.41)	16S rDNA, OmpA and gltA	*Rickettsiae*	cPCR	-	Sequencing performed but, no GeneBank submitted accession numbers
Selim et al., 2018	Menoufia, Qalubia, Alexandria and Gharbia	sheep<br>goats	2015–2016	110<br>80	37 (33.6)<br>13 (16.3)	icd	*C. burnetii*	qPCR	-	-
Selim et al., 2019	Alexandria	sheep	NS	21®<br>6<br>3	21<br>6<br>3	IS1111<br>-<br>-	*C. burnetii*	qPCR<br>MLVA<br>MST	-	-
Abbass et al., 2020	Assiut	sheep<br>goats	2016-2017	30*<br>18*	14 (46.7)<br>8 (44.4)	IS1111	*C. burnetii*	RT-qPCR	-	-
Abdel-Baky et al., 2020¥	NS	sheep	2014-2016	120	37.04%	OspA and flaB	*B. burgdorferi*	nPCR	-	-
Tumwebaze et al., 2020	Menoufia	sheep	2017	66	0 (0)<br>6 (9.1)	ssu rRNA<br>msp4	*B. ovis*<br>*A. ovis*	cPCR<br>cPCR	 4	 MN882167- MN882170
Abdullah et al., 2021a	variable	sheep<br>goats	2019-2020	38<br>28	3 (7.89)<br>1 (3.57)	ITS	*Bartonella sp.*	cPCR	-	-

Abdullah et al., 2021b	Giza, Beni-Suef, Qalyubia, Sinai	sheep	2016-2018	58	5 (8.6)	5.8S rRNA	*Piroplasmida*	qPCR		
					5 (8.6)	18S rRNA	*T. ovis*	nPCR		MN625886
					4 (6.9)	23S rRNA	*Anaplasmataceae*	qPCR		
					2 (3.4)	Ana-rpoB	*An. marginale*	cPCR		MN625934, MN624136
					1 (1.7)	Ana-rpoB	*An. ovis*	cPCR		MN624132
					1 (1.7)	Ana-rpoB	*An. platys-like*	cPCR		MN626398, MN624138
					2 (3.4)	16S rRNA	*Borrelia sp.*	qPCR		
					2 (3.4)	16S rRNA	*Bo. Theileri*	cPCR		MN621894
					1 (1.7)	IS1111	*Coxiella burnetii*	qPCR		
					0	gltA	*Rickettsia sp.*	qPCR		
					0	ITS3	*Bartonella sp*	qPCR		
		goats		33	1 (3)	IS1111	*Coxiella burnetii*	qPCR		
					0	5.8S rRNA	*Piroplasmida*	qPCR		
					0	23S rRNA	*Anaplasmataceae*	qPCR		
					0	16S rRNA	*Borrelia sp.*	qPCR		
					0	gltA	*Rickettsia sp.*	qPCR		
					0	ITS3	*Bartonella sp*	qPCR		
Al-Hosary et al., 2021a	Menoufia, Beheira, El-Wady El-Geded	sheep	2014	115	6 (5.22)	18S rRNA	*Theileria sp.*			AB986193 and AB986194
					5 (4.37)		*T. ovis*	cPCR	5	KY494648- KY494650
					1 (0.87)		*T. lestoquardi*	nPCR	1	KY494651
Barghash, 2022	Sinai	sheep	2020-2021	108	11(10.19)	BbSBP-4	*B. bovis*	cPCR	1	MZ197895
					22(20.37)	tams1	*T. annulata*		1	MZ197898
		goats		48	8(16.67)	BbSBP-4	*B. bovis*		1	MZ197894
					24(50.00)	tams1	*T. annulata*		1	MZ197897
Ben Said et al., 2022	Gharbia, Kafr Elsheikh, Menofia, Alexandria	sheep	2020	355	55 (15.5)	msp4	*A. ovis*	cPCR	4	OL859531 to OL859534

*Seropositive samples

placental cotyledons and vaginal discharges were examined from aborted animals.

®Vaginal swabs from aborted sheep

¥ Studies not included in Meta-analysis.

Abbreviations: **ama-1**; Apical membrane antigen 1 genes, **Ana-rpoB**; Anaplasma gene encoding the β-subunit of RNA polymerase, **BbSBP-4;** B. bovis Rhoptry Associated Protein-4; **IS1111**; C. burnetii transposon-like sequence, **msp4**; Merozoite surface protein 4, **icd**; iso citrate dehydrogenase gene, **MST;** Multi-spacer sequence typing, **MLVA,** Multiple-locus variable-number tandem repeat analysis, **rap-1**; Rhoptry-associated protein 1 gene, **SSU rRNA**; small subunit ribosomal RNA.

Supplementary Table S3: Study characteristics of tick-borne pathogens molecular surveys in equines from Egypt.

Reference	Region	Host	Study Year	Sample size	No. positive (%)	Marker	Species	Molecular tools	Sequenced isolates	Accession numbers
Mahdy et al., 2016	Giza and Cairo	horses<br>donkeys	NS	168<br>133	104 (61.9)<br>67 (50.4)	EMA-1	*T. equi*	nPCR	1	KX262963
Mahmoud et al., 2016	Giza	horses<br><br>donkeys	NS	88<br><br>51	32 (36.4)<br>17 (19.3)<br>22 (43.1)<br>8 (15.7)	ema-1<br>rap-1<br>ema-1<br>rap-1	*T. equi*<br>*B. caballi*<br>*T. equi*<br>*B. caballi*	nPCR	3	KR811095- KR811097
Kuraa and Nageib, 2017	Assiut	donkeys	NS	50	19 (38)	B1	*T. equi*	cPCR	-	-
El-Seify et al., 2018	Giza and Cairo	horses<br>donkeys<br>mules	2015-2016	45<br>50<br>5	11 (24.4)<br>18 (36)<br>1 (20)	18S rRNA	*T. equi*	cPCR	1<br>1<br>1	MF192854<br>MF192856<br>MF192855
El-Sayed et al., 2020	Menoufia, Mersa Matruh, Giza	donkeys	2017	149	9 (6.04)	EMA-1	*T. equi*	nPCR	1	LC269846
Al-Araby et al., 2021	Dakahlia	horses	NS	147	14 (9.52)	EMA-1	*T. equi*	nPCR	-	-
Abdullah et al., 2021a	variable	horses<br>donkeys	2019-2020	8<br>22	1 (12.5)<br>2 (9.09)	ITS	*Bartonella sp.*	cPCR	-	-
Elsawy et al., 2021	Cairo, Giza, Almonofia, Al fayoum, Beni Suef, Ismailia, and Alexandria	horses<br><br><br><br>donkeys	NS	79<br><br><br><br>76	16 (20.3)<br>1 (1.2)<br>42 (53.1)<br>3 (4.5)<br>10 (13.1)<br>0 (0.0)<br>29 (38.1)<br>18 (26.8)	18S rRNA	*T. equi*<br>*B. caballi*<br>*T. haneyi*<br>Mixed *Theleiria*<br>*T. equi*<br>*B. caballi*<br>*T. haneyi*<br>Mixed *Theleiria*	mPCR<br>mPCR<br>cPCR<br><br>mPCR<br>mPCR<br>cPCR	5<br>1<br>2<br><br>4<br>1<br>3	MW659071- MW659075<br>MW678758<br>MW591694, MW591695<br><br>MW659076 -MW659079<br>MW678759<br>MW591692, MW591693, MW591697

Abdullah et al., 2022	Cairo, Beni Suef	horses	2009	320	13 (4.1)	5.8S rRNA	Piroplasmida	qPCR		
					4 (1.3)	18S rRNA	*Th. equi*	nPCR	2	MN625897 and MN625898
					9 (2.8)	18S rRNA	*Th. sp. Africa*	nPCR	4	MN625900- MN625903
					0	gltA	Rickettsia sp.	qPCR		
					0	16S rRNA	Borrelia sp.	qPCR		
					0	IS1111	C. burnetii	qPCR		
					0	ITS3	Bartonella sp.	qPCR		
		donkeys		15	4 (26.6)	23S rRNA	Anaplasmataceae	qPCR		
					2 (13.3)	23S rRNA	*A. marginale*	cPCR	1	MN614103
					2 (13.3)	23S rRNA	*A. ovis*	cPCR	1	MN614104
					2 (13.3)	5.8S rRNA	Piroplasmida	qPCR		
					2 (13.3)	18S rRNA	*Th. ovis*	nPCR	2	MN625886 and MN625887
					0	gltA	Rickettsia sp.	qPCR		
					0	16S rRNA	Borrelia sp.	qPCR		
					0	IS1111	C. burnetii	qPCR		
					0	ITS3	Bartonella sp.	qPCR		

Abbreviations: **EMA-1**; T. equi merozoite antigen-1, **gltA**; Citrate synthase, **IS1111**; C. burnetii transposon-like sequence, **rap-1**; Rhoptry-associated protein 1 gene

Supplementary Table S4: Study characteristics of tick-borne pathogens molecular surveys in dromedary camels (*Camelus dromedarius*) from Egypt.

Reference	Region	Study Year	Sample size	No. positive (%)	Marker	Species	Molecular tools	Sequenced isolates	Accession numbers
Youssef et al., 2015	Giza	2009	30	21 (70)	SSU rRNA	*Theileria spp.*	cPCR	-	-
				18 (60)	30-KDa	*T. annulate*			

				3 (10)		*Theileria spp.*			
Abou El-Naga and Barghash, 2016	Matrouh	2012-2015	331	223 (67.37)<br>223 (67.37)<br>172 (51.96)<br>238 (71.9)<br>61 (18.43)<br>36 (10.87)<br>25 (7.55)	 msp1β<br>-<br>SSU rRNA<br>CPS II<br>SSU rRNA	*Anaplasma sp.*<br>*A. marginale*<br>*A. centrale*<br>*Th. Camelensis*<br>*Babesia sp.*<br>*B. bovis*<br>*B. bigemina*	cPCR	-	-
Abdullah et al., 2018	Cairo, Giza, and Sinai	2017	113	52 (46)	IS30A	*C. burnetii*	cPCR	-	-
Abd El-Baky and Allam, 2018	Variable	2014-2016	110	65 (50.39)<br>20<br>10<br>6<br>5<br>6<br>5<br>13	16S rRNA<br>msp4 and hsp60	***Anaplasmataceae***<br>*A. phagocytophilum*<br>*A. marginale*<br>*A. ovis*<br>*A. bovis*<br>*A. centrale*<br>*A. platys*<br>*Anaplasma spp.*	cPCR	-	Sequencing performed but, no GeneBank submitted accession numbers
Allam et al., 2018	Variable	2014-2016	120	66 (64.71)	16S rDNA, OmpA and gltA	*Rickettsiae*	cPCR	-	Sequencing performed but, no GeneBank submitted accession numbers
Abdullah et al., 2019a	Giza and Cairo	NS	112	19 (16.9)	IS1111	*C. burnetii*	qPCR	-	-
Abdullah et al., 2019b	Cairo, Giza and Sinai	2013-2014	61	25 (41)	OmpA and gltA	*Rickettsiae*<br>*R. africae*	cPCR		U83436.2
Abdel-Baky et al., 2020¥	NS	2014-2016	110	40.74%	OspA and flaB	*B. burgdorferi*	nPCR	-	-
Abdullah et al., 2021b	Cairo, Giza, Sinai	2016-2018	149	10 (6.7)<br>1 (0.7)<br>1 (0.7)<br>8 (5.4)<br>0<br>0<br>0<br>0<br>0	23S rRNA<br>Ana-rpoB<br>Ana-rpoB<br>Ana-rpoB<br>5.8S rRNA<br>16S rRNA<br>gltA<br>IS1111<br>ITS3	***Anaplasmataceae***<br>*An. marginale*<br>*An. platys*<br>*An. platys-like*<br>*Piroplasmida*<br>*Borrelia sp.*<br>*Rickettsia sp.*<br>*C. burnettii*<br>*Bartonella sp*	qPCR<br>cPCR<br>cPCR<br>cPCR<br>qPCR<br>qPCR<br>qPCR<br>qPCR<br>qPCR		 MN625938<br>MN625937<br>MN626396, MN626401

El-Sayed et al. 2021 Rizk et al., 2021	Halayeb and Shalateen	2017	142	4 (2.81)	RAP-1	*B. bovis*	nPCR	1	MF737083
				17 (11.97)	SSU rRNA	*Babesia microti*	nPCR	1	MF737082
Mohamed et al., 2021	Abu Simbel	NS	100	29 (29)	16S rDNA	***Anaplasmataceae***	cPCR	**28**	LC592622-LC592650
					groEL	*Ca. An. camelii*		**10**	LC592667- LC592677
					gltA			**10**	LC592678-LC592688

Abbreviations: **Ana-rpoB**; Anaplasma gene encoding the β-subunit of RNA polymerase, **CPS II**; gene encoding the enzyme carbamoyl phosphate synthetase II, **gltA**; Citrate synthase, **groEL**; 'heatshock operon' **IS1111**; C. burnetii transposon-like sequence, **msp1β**; Major surface protein–1β encoding gene, **msp4**; Merozoite surface protein 4, **ompA**; Outer membrane protein A, **rap-1**; Rhoptry-associated protein 1 gene, **SSU rRNA**; small subunit ribosomal RNA.

¥ Studies not included in Meta-analysis

Supplementary Table S5: Study characteristics of tick-borne pathogens molecular surveys in dogs from Egypt.

**Reference**	**Region**	**Study Year**	**Sample size**	**No. positive (%)**	**Marker**	**Species**	**Molecular tools**	**Sequenced isolates**	**Accession numbers**
Elhelw et al., 2014	NS	2008-2009	26	6 (23)	OspA	*B. burgdorferi*	cPCR	1	KC522030
Salem and Farag, 2014	Giza	2011-2012	13*	13	18S rRNA	*B. vogeli*	nPCR	-	-
El-Neshwy et al., 2020	Sharkia and Dakahlia	2018-2019	75	14 (18.67)	18s rRNA	*B. vogeli*	nPCR	-	-
Abdullah et al., 2021b	Cairo	2016-2018	203	1 (0.5)	5.8S rRNA	*Piroplasmida*	qPCR		
				1 (0.5)	18S rRNA	*Ba. canis*	nPCR		MN625891

				7 (3.4)	23S rRNA	*Anaplasmataceae*	qPCR		
				7 (3.4)	Ana-rpoB	*An. platys*	cPCR		MN625936, MN626395, MN624140
				3 (1.5)	gltA	*Rickettsia sp.*	qPCR		
				3 (1.5)	OmpB	*Rickettsia africae-like*	nPCR		MN629892, MN629893
				0	IS1111	*C. burnettii*	qPCR		
				0	ITS3	*Bartonella sp*	qPCR		
				0	16S rRNA	*Borrelia sp.*	qPCR		
Elhelw et al., 2021	Giza Cairo	2017	100	1 (1.67)	16S rRNA	*B. burgdorferi*	cPCR	1	MH685928
El-Dakhly et al., 2021¥	Alexandria	NS	70	14 (20)	16sRNA	*Anaplasma/Ehrlichia*	cPCR	-	-
Selim et al., 2021b	Cairo, Giza, Qalyubia, Gharbia and Kafr El Sheikh	2019-2020	500	32 (6.4)	16S rRNA	*A. platys*	cPCR	1	LC632659
Selim et al., 2021c	Cairo, Giza, and Qalyubia	NS	400	39 (9.7)	16S rRNA	*E. canis*	cPCR	1	LC490598
Zaki et al., 2021	Cairo	2017-2019	242	62 (25.62)	18S rRNA	*B. vogeli*	cPCR	1	MT565474
Izenour et al., 2022	Cairo	2019	116	1	groEL	*Anaplasma sp.*	cPCR	1	MW557303
				1	18S rRNA	*Babesia sp.*		1	MW556745
				2	16S rRNA	*Ehrlichia sp.*			
				1	16S rRNA	*Borrelia sp.*			
				0	ssrA	*Bartonella sp.*			
Selim et al., 2022a	Giza, Kafr El Sheikh, Qalyubia and Gharbia	2019	275	14 (5.1)	18S rRNA	*B. vogeli*	cPCR	1	LC651125

*Samples from dogs with clinical signs of babesiosis and also were positive by microscopy.

¥ Studies not included in Meta-analysis

Abbreviations: **Ana-rpoB**; Anaplasma gene encoding the β-subunit of RNA polymerase, **gltA**; Citrate synthase, **groEL**; 'heatshock operon' **ompB**; Outer membrane protein B, **ospA**; Outer surface protein A. gene.

Supplementary Table S6: Study characteristics of molecular surveys of pathogens in ticks infesting livestock animals from Egypt.

Reference #	Host	No. infested (%)	Tick species (N.) ®	Pathogens	Marker	No. positive (%)
Adham et al., 2009[1]	NS	-	Boophilus annulatus	*B. bovis*<br>*B. bigemina*	SSrRNA	55/100 (55)<br>66/100 (66)
Adham et al., 2010[1]	NS	-	B. annulatus<br>H. dromedarii<br>H. excavatum<br>R. sanguineus<br>A. lepidum<br>O. savignyi	*B. burgdorferi*	OspC	4/50 (8)<br>7/28 (25)<br>12/24 (50)<br>2/23 (8)<br>4/14 (28)<br>31/47 (66)
Allam et al., 2018[1]	Camels<br>Cattle<br>Sheep	33.62%<br>59.03%<br>7.35%	A. gemma<br>A. lepidum<br>A. variegatum<br>B. annulatus<br>H. albiparmatum<br>H. a. excavatum<br>H. dromedarii<br>H. impeltatum<br>H. m. rufipes<br>H. m. marginatum<br>H. truncatum<br>Rh. humeralis<br>Rh. pulchellus<br>Rh. sanguineus	*Rickettsiae* sp.<br>R. aeschlimannii-like<br>R. africae-like<br>R. sibrica mongolitimonae-like	16S rDNA, OmpA and gltA	15/51 (4.78)<br>20/67 (6.37)<br>5/55 (1.59)<br>5/2922 (1.59)<br>6/25 (1.91)<br>24/127 (7.64)<br>112/1288 (35.67)<br>24/91 (7.64)<br>24/53 (7.64)<br>24/105 (7.64)<br>16/90 (5.10)<br>8/20 (2.55)<br>12/22 (3.82)<br>19/307 (6.05)
Chisholm et al., 2012[1]¥	Cattle, buffalo, sheep, camels	NS	Hyalomma (138 pools)<br>H. excavatum<br>H. dromedarii	CCHFV	RT-PCR-S segment	6/138 (4.34)<br>1<br>5
Elhelw et al., 2014	cattle	NS	H. anatolicum excavatum	*B. burgdorferi*	OspA	3/14 (21.4)
ELHariri et al., 2017¥	buffaloes	NS	Rhipicephalus (280), Boophilus (150), H. an. anatolicum (30), and H. a. excavatum (40).	*A. marginale*	msp1α	130/150 (86.6)

Hassan et al., 2017	cattle		R. annulatus	*B. theileri*<br>*B. bigemina*	flab<br>18S rRNA	10/172 (5.8)<br>2/172 (1.16)
	camels		O. savignyi	*B. theileri*	flab	6/611 (1)
Abdel-Baky et al., 2020[1]¥	camels, cattle, sheep		A. variegatum<br>A. lepidum<br>B. annulatus	*B. burgdorferi*	OspA and flaB	43.24%<br>30.63%<br>26.13%
AL-Hosary et al. 2021b¥	cattle	NS	H. excavatum	*Theileria annulata*<br>*Babesia occultans*<br>*An. marginale*<br>*Anaplasma platys*<br>*Midich. mitochondrii*<br>*Ehrl. chaffeensis-like*<br>*Ehrlichia minasensis*	RLB and variable targets	18.1 %<br>1.8 %<br>28.5 %<br>0.25 %<br>11.6 %<br>1.8 %<br>1 %
			R. annulatus	*T. annulata*<br>*B. bovis*<br>*A. marginale*<br>*A. platys*<br>*M. mitochondrii*<br>*E. minasensis*		2.3 %<br>0.6 %<br>18.0 %<br>1.2 %<br>2.9 %<br>0.6 %
Abdullah et al., 2022	equines	NS	R. annulatus (61)	Anaplasmataceae<br>*A. platys-like*<br>*A. marginale*<br>*Candidatus E. rustica*<br>*Ehrlichia sp.*<br>*Borrelia sp.*<br>*B. theileri*	23S rRNA<br>23S rRNA<br>23S rRNA<br>23S rRNA<br>23S rRNA<br>16S rRNA<br>16S rRNA	9 (14.7)<br>1 (1.6)<br>1 (1.6)<br>4 (6.6)<br>3 (4.9)<br>2 (3.3)<br>2 (3.3)
Abdel-Shafy et al., 2012	camels	10	H. dromedarii (42)<br>H. impeltatum (15)<br>H. marginatum (5)	*R. africae and R. aeschlimannii*	gltA ompA, ompB, Sca4	8 (19.04)<br>15 (100)<br>1 (20)
Abdullah et al., 2016	camels	NS	H. marginatum (5)	*Rickettsia* sp.	OmpA and gltA	1 (20)
Barghash et al., 2016¥	camels	212 (85.14)	H. dromedarii (73.65%), H. rufipes (12.03%), H. truncatum (6.62%), H. anatolicum excavatum (4.73%), H. impeltatum	*T. evansi, T. brucei, B. bovis, B. bigemina and Theileria sp., A. marginale, Histophilus somni, Pasteurella multocida and*	Variable	NS

			(1.62%), and others (1.35%).	*Mycoplasma spp.*		
Abdullah et al., 2018	camels	NS	H. dromedarii (177)	*C. burnetii*	IS30A	10/190 (5.3)
Abdullah et al., 2019b	camels	NS	Hayalomma spp. (99)<br>H. marginatum (5/99)	*Rickettsia spp.* (*R. africae*)	OmpA and gltA	1/99 (1.01)<br>1/5 (20)
Ghoneim et al., 2020	camels	181 (100)	H. dromedarii (259)<br>A. variegatum (18)<br>H. anatolicum anatolicum (19)<br>R. pulchellus (28)	*C. burnetii*	IS1111	17 (6.6)<br>1 (5.6)<br>1 (5.3)<br>1 (3.6)
Bendary et al., 2022	camels	NS	H. dromedarii (837 pools)<br>H. rufipes (411 pools)	CCHFV	RT-PCR-S segment	11 (1.3)<br>7 (1.7)
Ghafar and Amer, 2012	dogs	NS	R. sanguineus (401)	A. phagocytophilum	16S rRNA	55/401 (13.7)
Elhelw et al., 2014	dogs	NS	R. sanguineus (12)	*B. burgdorferi*	OspA	7 (58.3)
Abdullah et al., 2016	dogs	NS	R. sanguineus (597)	*Rickettsia* sp.	OmpA and gltA	4/71 (5.6)
Nasr et al., 2020	dogs	156	R. sanguineus (156 pools)	*A. platys*<br>*E. canis*	16S rRNA	2 (1.32)<br>3 (1.98)
Elhelw et al., 2021	dogs	69 (69)	R. sanguineus (60)	*B. burgdorferi*	16S rRNA	1 (1.67)

The collected ticks were morphologically identified using published identification keys and additionally some molecular markers were used.

® Tick species and genera examined for pathogens using molecular markers

[1] Theses articles identified pathogens in ticks' pools collected from various hosts

¥ Studies were not included in Meta-analysis

Abbreviated Tick genus: **A.**; Amblyomma, **D.**; Dermacentor, **H.**; Hyalomma, **R.**; Rhipicephalus, **O.**; Ornithodoros

Abbreviations: **flaB**; encoding flagellin B, **gltA**; Citrate synthase gene, **groEL**; 'heatshock operon', **IS1111**; *C. burnetii* transposon-like sequence, **ompA**; Outer membrane protein A, **ompB**; Outer membrane protein B, **ospA**; Outer surface protein A. gene, **Sca4**; Cell surface antigen-4 (gene D).

Supplementary Table S7: Haplotypes of *T. annulata* Tams-1 isolates from ruminant animals in Egypt.

Haplotype	Accession number	Host	Region
Ta-1	**AB917277.1**	Cattle	Menoufia
Ta-2	**AB917296.1**	Cattle	Menoufia
Ta-3	**AB917298.1**	Cattle	Menoufia
Ta-4	**AB917284.1**	Cattle	Behera
	**AB917285.1**	Cattle	Giza
	**AB917293.1**	Cattle	Menoufia
Ta-5	**AB917287.1**	Cattle	Giza
Ta-6	**KJ021629.1**	Cattle	El-Fayoum
Ta-7	**MN251047.1**	Cattle	Sharkia
	**MN251046.1**	Buffalo	Sharkia
	**MZ197896.1**	Cattle	Sinai
	**MZ197898.1**	Sheep	Sinai
Ta-8	**AB917294.1**	Cattle	Menoufia
	**AB917295.1**	Cattle	Menoufia
Ta-9	**KJ021627.1**	Cattle	Assiut
Ta-10	**KF765518.1**	Cattle	Port-Said
Ta-11	**KF765519.1**	Cattle	Port-Said
Ta-12	**AB917281.1**	Cattle	Behera
Ta-13	**AB917286.1**	Cattle	Giza
Ta-14	**AB917289.1**	Cattle	Giza
Ta-15	**AB917278.1**	Cattle	Menoufia
Ta-16	**AB917280.1**	Cattle	Behera
Ta-17	**AB917283.1**	Cattle	Behera
Ta-18	**AB917300.1**	Cattle	Menoufia
Ta-19	**MZ197897.1**	Goat	Sinai
Ta-20	**AB917290.1**	Cattle	Giza
Ta-21	**AB917275.1**	Cattle	Menoufia
Ta-22	**AB917288.1**	Cattle	Giza

Ta-23	**AB917276.1**	Cattle	Menoufia
Ta-24	**AB917297.1**	Cattle	Menoufia
Ta-25	**AB917279.1**	Cattle	Behera
	**AB917282.1**	Cattle	Behera
	**AB917291.1**	Cattle	Behera
	**AB917292.1**	Cattle	Behera
Ta-26	**AB917301.1**	Cattle	Menoufia
Ta-27	**AB917302.1**	Cattle	Menoufia
Ta-28	**MH796634.1**	Cattle	New Valley
	**LC549654.1**	Cattle	NS
Ta-29	**KJ021628.1**	Cattle	New Valley
Ta-30	**MH796632.1**	Cattle	New Valley
Ta-31	**AB917299.1**	Cattle	Menoufia
Ta-32	**KJ021626.1**	Cattle	El-Fayoum
Ta-33	**MH796633.1**	Cattle	New Valley
	**LC549653.1**	Cattle	NS
